# Susceptibility of sweet potato (*Ipomoea batatas*) peel proteins to digestive enzymes

**DOI:** 10.1002/fsn3.110

**Published:** 2014-04-01

**Authors:** Katherine P Maloney, Van-Den Truong, Jonathan C Allen

**Affiliations:** 1 Department of Food, Bioprocessing, and Nutrition Sciences, North Carolina State UniversityRaleigh, North Carolina; 2 USDA-ARS, SAA Food Science Research Unit, North Carolina State UniversityRaleigh, North Carolina

**Keywords:** Amylase, amylase inhibitor, digestibility, protease, sweet potato peel, trypsin inhibitor

## Abstract

Sweet potato proteins have been shown to possess antioxidant and antidiabetic properties in vivo. The ability of a protein to exhibit systemic effects is somewhat unusual as proteins are typically susceptible to digestive enzymes. This study was undertaken to better understand how digestive enzymes affect sweet potato proteins. Two fractions of industrially processed sweet potato peel, containing 6.8% and 8.5% protein and 80.5% and 83.3% carbohydrate, were used as a source of protein. Sweet potato proteins were incubated with pepsin, trypsin, and chymotrypsin and protein breakdown was visualized with SDS-PAGE. After pepsin digestion, samples were assayed for amylase inhibitory activity. Sporamin, the major storage protein in sweet potatoes, which functions as a trypsin inhibitor as well, exhibited resistance to pepsin, trypsin, and chymotrypsin. Sporamin from blanched peel of orange sweet potatoes was less resistant to pepsin digestion than sporamin from outer peel and from extract of the white-skinned Caiapo sweet potato. Trypsin inhibitory activity remained after simulated gastric digestion, with the Caiapo potato protein and peel samples exhibiting higher inhibitory activity compared to the blanched peel sample. Amylase and chymotrypsin inhibitory activity was not present in any of the samples after digestion.

## Introduction

Sweet potato proteins possess numerous nutraceutical properties. Caiapo, currently called Caiapo Potato Powder, is an extract from a white-skinned sweet potato cultivar (originally Cayapo) marketed as an antidiabetic supplement and has been shown to lower fasting blood glucose levels and increase insulin sensitivity in type II diabetics (Ludvik et al. 2003, 2004a,b; Kusano et al. 2005). In vitro, sweet potato trypsin inhibitor proteins have been shown to possess antioxidant properties with scavenging abilities against 2,2-diphenyl-1-picrylhydrazyl (DPPH), hydroxyl radical (Hou et al. 2001, 2005), and reactive nitrogen species (Huang et al. 2007a). They have also been shown to increase serum superoxide dismutase, catalase, and glutathione peroxidase activity in mice (Huang et al. 2008). Anti-proliferative properties (NB4 promyelocytic leukemia cells in vitro) have also been reported (Huang et al. 2007b).

Multiple proteins isolated from sweet potatoes have been shown to possess trypsin inhibitory activity (Sugiura et al. 1973; Obidairo and Akpochafo 1984; Hou and Lin 1997; Jaw et al. 2007); however, the protein found in the greatest quantity that possesses trypsin inhibitory activity is the 25 kDa storage protein, sporamin. Sporamin makes up over 80% of the total protein found in sweet potatoes (Maeshima et al. 1985). Trypsin inhibitory activity has been shown to vary by cultivar. Bradbury et al. (1984) found a 67-fold range in trypsin inhibitory activity, from 0.33 TIU to 22.1 TIU, among sweet potato cultivars from the Highlands of Papua New Guinea. Nutraceutical properties of the trypsin inhibitors have also been shown to vary by cultivar. Hou et al. (2005) found that trypsin inhibitors isolated from the cultivar Tainong 65 exhibited a higher degree (threefold) of protection against Cu^2+^-induced human LDL peroxidation than trypsin inhibitors isolated from the cultivar Tainong 57. Trypsin inhibitors from Tainong 57, however, exhibited a higher degree (10-fold) of protection against hydroxyl radical-induced DNA damage of calf thymus compared to trypsin inhibitors isolated from Tainong 65. Trypsin inhibitors are stable over a wide pH range (Sugiura et al. 1973), but their activity can be reduced by processing (Obidairo and Akpochafo 1984; Zhang and Corke 2001; Kiran and Padmaja 2003).

While less studied, amylase inhibitors have also been identified in sweet potatoes with amylase inhibitory activity varying by cultivar (Rekha et al. 1999). The stability of the amylase inhibitors to processing has been found to vary by cultivar (Rekha and Padmaja 2002). Amylase inhibitors, in general, have been investigated for their potentially positive impact on diabetes management and weight control, due to their ability to slow and/or reduce starch digestion, thus slowing and/or reducing glucose absorption, lowering glycemic response, and possibly lowering caloric absorption. In order to function as an inhibitor of intestinal digestive enzymes in vivo, a protein must survive gastric digestion. Several studies found high in vitro inhibitory activities, but then failed to find the same inhibitory activities in vivo (Bo-Linn et al. 1982; Carlson et al. 1983), possibly due to susceptibility of the inhibitor to digestion. Therefore, in this study, extracts were subjected to in vitro gastric digestion prior to assaying for amylase inhibitory activity.

In order to have systemic effects, such as the effects seen for Caiapo Potato Powder administration, proteins must survive both gastric and intestinal digestion, so that they will have the chance to be absorbed. The first step in determining if protein extracts from orange-flesh cultivars of sweet potato have equivalent antidiabetic activity to the extract from white-skinned Caiapo sweet potatoes is to ensure proteins can be absorbed intact by the body. A limitation to the use of proteins for therapeutic treatment is their susceptibility to digestive enzymes (Goldberg and Gomez-Orellana 2003); however, if the protein can remain intact in the presence of digestive enzymes, absorption may occur. Several proteins have been shown to pass through the intestinal barrier intact (Castell et al. 1997; Seno et al. 1998; Imai et al. 2002). The objectives of this study were to determine if proteins were present in sweet potato extracts that were resistant to digestive enzymes and if activities were retained after in vitro digestion, in order to better understand how sweet potato proteins could exhibit systemic effects in the body. Caiapo, a sweet potato protein supplement already on the market, was compared to extracts from two other potential sources for sweet potato protein supplements.

## Materials and Methods

### Materials

Enzymes were obtained from Sigma-Aldrich (St. Louis, MO). The reported activity for pepsin from porcine gastric mucosa was 3802 units/mg protein, *α*-chymotrypsin from bovine pancreas was 59.3 units/mg protein, and trypsin from porcine pancreas was 14476 BAEE units/mg protein. Soluble starch was obtained from Sigma-Aldrich. Caiapo potato powder was obtained from Fuji-Sangyo Company, Ltd. (Kagawa, Japan). Sweet potato peel was obtained from a local processing plant (Yamco, LLC, Snow Hill, NC). Peel was from a mixture of orange-flesh cultivars including Beauregard, Jewel, and Covington. Peel was obtained from two different points along the processing line. Material was obtained from the initial peeling of the sweet potatoes before any further processing, from hereafter referred as “peel,” and material was obtained from a secondary peeling after blanching of the sweet potatoes, from hereafter referred to as “blanched peel.” Upon receipt, the peel and blanched peel were freeze-dried and stored at −20°C. The freeze-dried material was compared with peel extract prepared as previously described (Maloney et al. 2012), but the unprocessed freeze-dried peel was closer in composition to the Caiapo Potato Powder, so the unprocessed freeze-dried peel and blanched peel were used for these experiments.

### Composition of peelings

Proximate analysis for fat, carbohydrate, dietary fiber, crude fiber, protein, moisture, and ash was determined on samples of the peel and blanched peel by a contract laboratory (Microbac Laboratories, Inc., Warrendale, PA) using the AOAC methods listed in Table 1. Total phenol in the peel and blanched peel was determined by the method of Singleton et al. (1999).

**Table 1:** Proximate analysis of sweet potato peel and blanched peel

Analysis	Primary peel^1^ (%)	Primary peel (% DM)^2^	Blanched peel^1^ (%)	Blanched peel (% DM)^2^	Method
Fat (Mojonnier)	2.33–2.65	2.61	1.35–1.28	1.37	AOAC 922.06
Carbohydrate—total	77.0–76.4	80.5	79.9–80.4	83.3	Calculation^3^
Dietary Fiber—total	56.1–55.2	58.4	30.1–29.6	31.0	AOAC 991.43
Fiber—crude	22.4–22.3	23.5	14.2–14.0	14.7	AOAC 978.10
Protein^4^	6.40–6.49	6.77	8.11–8.20	8.48	AOAC 992.15 MOD
Moisture	4.76–4.74	0	4.01–3.65	0	AOAC 925.09
Ash	9.47–9.70	10.1	6.60–6.45	6.78	AOAC 923.03

1 Data show range of duplicate analyses.

2 Mean of duplicate samples converted to dry matter basis.

3 Carbohydrate % = 100 − (fat + protein + moisture + ash).

4 Protein factor, 6.25, AOAC 992.15 MOD.

### In vitro gastric digestion

The low-protease digestion assay of Mandalari et al. (2009) was followed with some modifications. Samples containing 1 g of freeze-dried peel, freeze-dried blanched peel, or Caiapo dissolved in 10 mL of 150 mmol/L NaCl, pH = 2, were incubated at 37°C for 10 min. A 100-*μ*L portion of pepsin solution (5 mg/mL, pH = 2) was then added to each sample. Samples were incubated at 37°C for 1 h with 100-*μ*L portions removed at 0, 1, 2, 5, 10, 30, and 60 min. The reactions were stopped by addition of 20 *μ*L 0.5 mol/L NaOH.

### In vitro duodenal digestion

The low-protease digestion assay of Mandalari et al. (2009) was again followed with some modifications. After simulated gastric digestion for 60 min, the pH of the protein solutions was adjusted to seven using 0.5 mol/L NaOH. A 100 *μ*L portion of either trypsin solution (25 *μ*g/mL, pH = 7) or chymotrypsin solution (0.5 mg/mL, pH = 7) was then added to 5 mL of each protein solution. Solutions were incubated at 37°C for 1 h with 100 *μ*L portions removed at 0, 1, 2, 5, 10, 30, and 60 min. The reactions were stopped by addition of 20 *μ*L SigmaFAST (Sigma-Aldrich, St. Louis, MO) protease inhibitor tablet solution (one tablet dissolved in 10 mL DI water).

### Amylase activity assay

The method of Bernfeld (2009) with some modifications was used to determine the amylase activity of the peel, blanched peel, and Caiapo before and after digestion, and to determine if amylase inhibitors were present in the extracts after digestion. A 1% soluble starch solution was prepared by dissolving 1 g soluble starch in 100 mL warm 20 mmol/L sodium phosphate buffer containing 150 mmol/L NaCl, pH = 7. For the assay of amylase activity, a 250 *μ*L portion of each protein solution before and after in vitro gastric digestion for 60 min was combined with 250 *μ*L sodium phosphate buffer, so that the final reaction buffer consisted of 20 mmol/L sodium phosphate buffer containing 150 mmol/L NaCl, pH = 7. For the assay of amylase inhibitory activity after gastric digestion, a 250 *μ*L portion of each protein solution after in vitro gastric digestion for 60 min was combined with 250 *μ*L sodium phosphate buffer containing porcine pancreatic amylase, so that the final reaction buffer consisted of 20 mmol/L sodium phosphate buffer containing 150 mmol/L NaCl, pH = 7. Amylase activity of the samples containing digested peel, blanched peel, or Caiapo in combination with porcine pancreatic amylase were compared to a sample containing only porcine pancreatic amylase. All solutions were incubated at 37°C for 15 min and then 1 mL of soluble starch solution was added to the protein solutions to start the reaction. Samples were incubated at 37°C for 3 min and then the reaction was stopped by the addition of 2 mL of 3,5-dinitrosalycylic acid reagent. Absorbance was measured at 540 nm. One unit of amylase activity was defined as the amount required to liberate 1 *μ*mol maltose per minute under the conditions of the assay.

### Gel electrophoresis

Changes in proteins over time during incubation with pepsin, trypsin, and chymotrypsin were visualized with reducing SDS-PAGE. Laemmli sample buffer and 10× tris/glycine/SDS running buffer were obtained from Bio-Rad (Hercules, CA), *β*-mercaptoethanol was obtained from Sigma-Aldrich, and SeeBlue Plus2 protein standard was obtained from Invitrogen (Carlsbad, CA). Sample and sample buffer (containing 1% *β*-mercaptoethanol) were combined in a 1:1 ratio and the mixture was heated for 5 min at 85°C. Samples were run on a 15% tris-HCl Ready Gel (Bio-Rad Laboratories) at constant voltage (200 V). Gels were stained with Imperial Protein Stain (Thermo Fisher Scientific, Rockford, IL). Band density was quantified with UN-SCAN-IT gel analysis software (Silk Scientific, Inc., Orem, UT).

### Statistical analysis

Data were analyzed by ANOVA using the software JMP (Version 9, SAS, Inc., Cary, NC). Mean differences were determined with Tukey–Kramer test (*P* < 0.05).

## Results and Discussion

### Composition of peelings

Proximate analysis for fat, carbohydrate, dietary fiber, crude fiber, protein, moisture, and ash from duplicate samples of peel and blanched peel are shown in Table 1. The major component (approximately 80% on a dry matter basis) of both samples was carbohydrate, which included 58.4% and 31.0% dietary fiber in the peel and blanched peel, respectively. Insoluble crude fiber contributed about half of the total dietary fiber. The remaining portion of the total carbohydrate is unidentified. An iodine test for starch was negative. Total phenol measurement found 6.1 and 1.4 mg/g of gallic acid equivalents in the peel and blanched peel, respectively. The protein content was 6.77% and 8.48% of the dry matter in the peel, and blanched peel, respectively (Table 1).

### Susceptibility of sporamin to digestive enzymes

**Figure 1:**
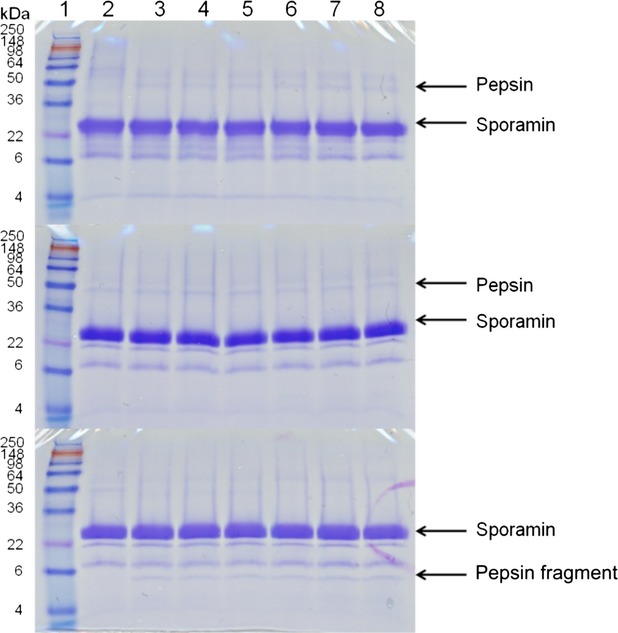
SDS-PAGE of Caiapo digested with pepsin (top), then with trypsin (middle), or chymotrypsin (bottom). The pepsin or pepsin fragment bands indicate the activity of the trypsin or chymotrypsin to the resistant sporamin or nonresistent (pepsin) proteins. Lane 1 contains SeeBlue Plus2 PreStained Standard. Lanes 2–8 contain samples in which the reaction was stopped at 0, 1, 2, 5, 10, 30, and 60 min, respectively.

**Figure 2:**
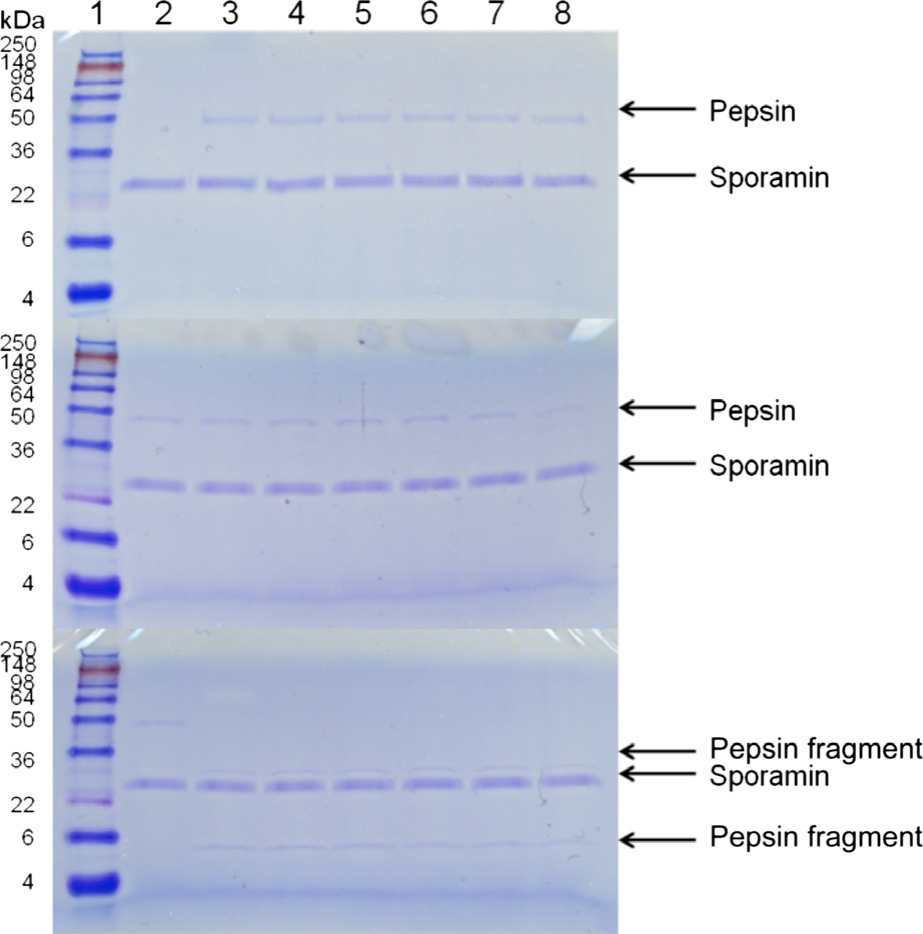
SDS-PAGE of sweet potato peel digested with pepsin (top), then with trypsin (middle), or chymotrypsin (bottom). Lane 1 contains SeeBlue Plus2 PreStained Standard. The pepsin or pepsin fragment bands indicate the activity of the trypsin or chymotrypsin to the resistant sporamin or nonresistent (pepsin) proteins. Lanes 2–8 contain samples in which the reaction was stopped at 0, 1, 2, 5, 10, 30, and 60 min, respectively.

**Figure 3:**
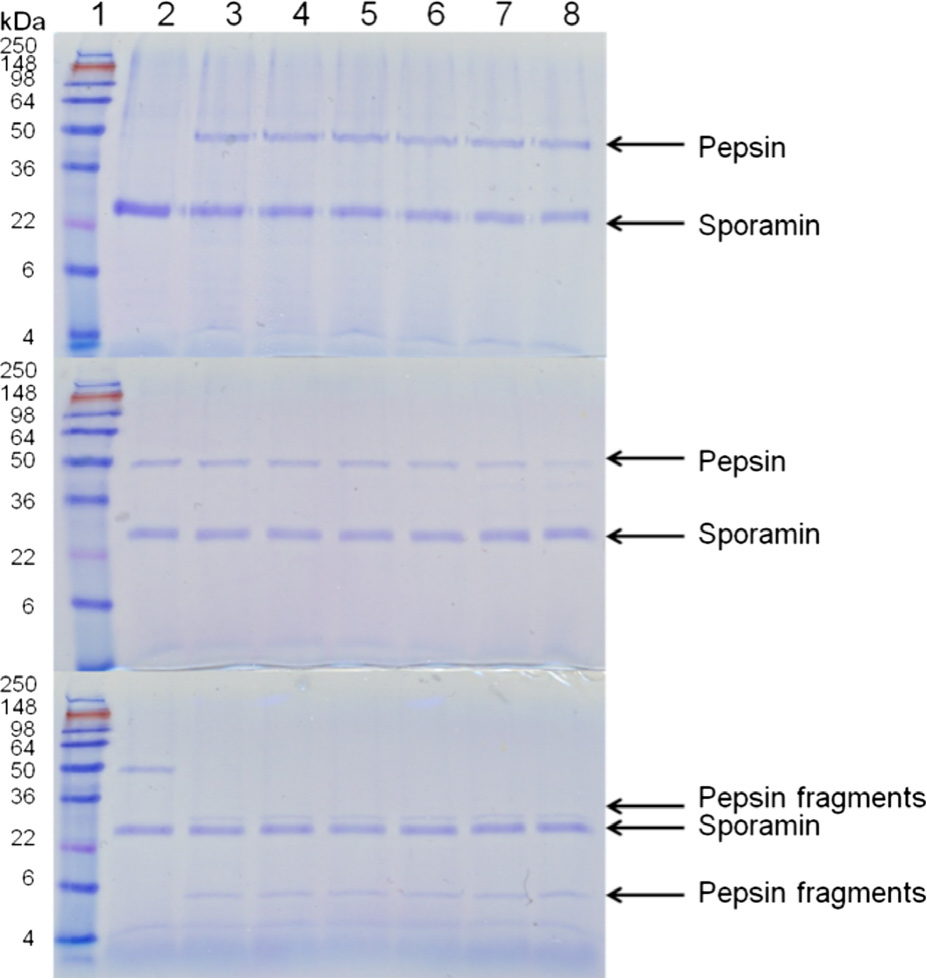
SDS-PAGE of blanched sweet potato peel digested with pepsin (top), then with trypsin (middle), or chymotrypsin (bottom). The pepsin or pepsin fragment bands indicate the activity of the trypsin or chymotrypsin to the resistant sporamin or nonresistent (pepsin) proteins. Lane 1 contains SeeBlue Plus2 PreStained Standard. Lanes 2–8 contain samples in which the reaction was stopped at 0, 1, 2, 5, 10, 30, and 60 min, respectively.

**Figure 4:**
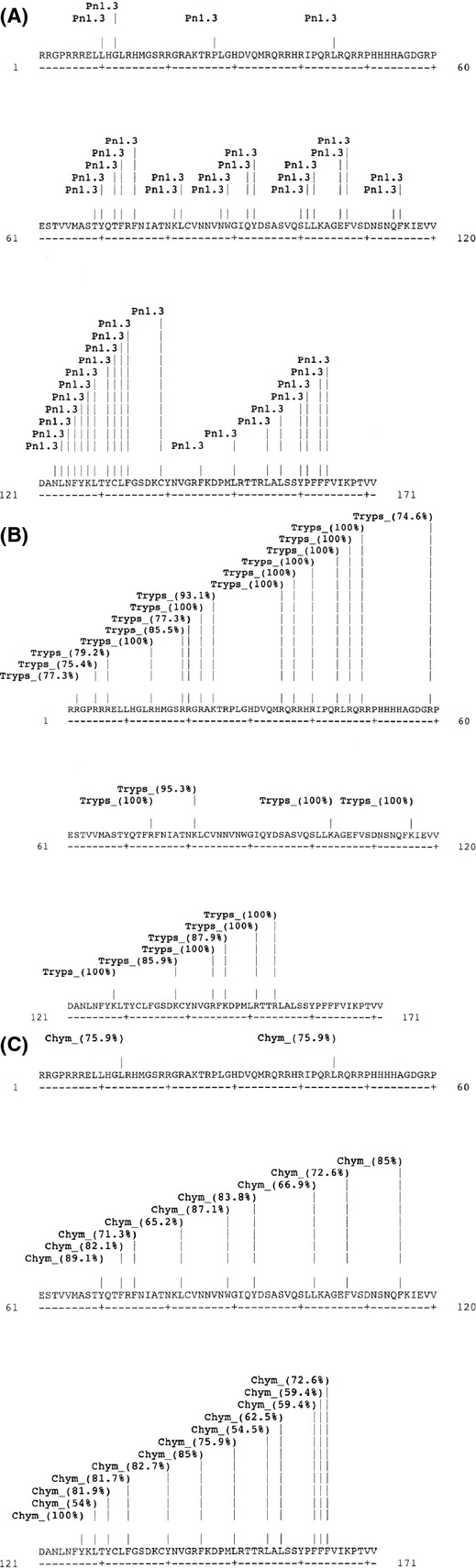
Theoretical (A) pepsin, (B) trypsin, or (C) chymotrypsin, cleavage sites of sporamin determined by inputting sporamin protein sequence from GenBank accession AAB52550 into ExPASy PeptideCutter. Likelihood of cleavage is shown in parenthesis. Only sites with a likelihood of cleavage greater than 50% are shown.

Visualization of proteins with SDS-PAGE at various points during digestion of Caiapo, peel extract, and blanched peel extract with pepsin, trypsin, and chymotrypsin revealed that sporamin exhibits resistance to cleavage by these enzymes. Our control protein, bovine serum albumin, on the other hand, was hydrolyzed after 1 min of incubation under the conditions of the assay (data not shown). The density of the sporamin band in Caiapo and peel extract remained unchanged despite incubation with pepsin, trypsin, and chymotrypsin (Figs. 1, 2). The density of the sporamin band in blanched peel extract decreased as incubation time increased, however, resistance to digestive enzymes was still noted (Fig. 3). After 1 min of incubation with pepsin, sporamin band density in the blanched peel sample decreased to 55% of the original band density. After 60 min, the band density decreased to 23% of the original density. The sporamin that remained after 60 min of incubation with pepsin was resistant to digestion by trypsin and chymotrypsin, indicated by the lack of difference between the sporamin band density at the start of incubation and after 60 min of incubation. Protein resistance to digestion might be due to either a unique amino acid sequence that hinders the ability of digestive enzymes to recognize cleavage sites, or compact structure, which hinders the ability of the digestive enzymes to reach the cleavage sites. The computer program ExPASy PeptideCutter was used to predict potential cleavage sites on sporamin for pepsin, trypsin, and chymotrypsin, in order to determine if resistance was due to a unique amino acid sequence. The sequence used for sporamin was GenBank accession AAB52550, determined by Yeh et al. (1997). Sporamin contained numerous potential cleavage sites for pepsin, trypsin, and chymotrypsin (Fig. 4, A–C), indicating that a unique amino acid sequence is not likely the mechanism for the resistance of sporamin to digestion. The compact structure of the protein is more likely responsible for the lack of cleavage by digestive enzymes. Sugiura et al. (1973) found that sweet potato trypsin inhibitors with molecular weights of 23 and 24 kDa (probably sporamin) were stable at pH = 2 and 37°C. As this is within the pH range and temperature of the stomach, it is likely that potential cleavage sites were inaccessible to pepsin due to the structural stability of sporamin. Compact structure has been found to play a role in reducing the digestibility of several proteins, including chickpea albumin (Clemente et al. 2000), lupin *γ*-conglutin (Capraro et al. 2009), and many allergenic albumins (Moreno and Clemente 2008).

In contrast to our findings, Hou et al. (2005) reported that free amino ends increased during incubation of the major root storage protein isolated from the sweet potato cultivars Tainong 57 and Tainong 65 with pepsin and chymotrypsin. The digestion procedure used by Hou et al. (2005) was more exhaustive than our procedure (incubation with pepsin for 8 h and then chymotrypsin for 4, 8, or 12 h), however, which could account for the difference in results. Hou et al. (2005) noted that the susceptibility to digestion of the protein did not hinder its antioxidant properties. In fact, scavenging ability against DPPH radical increased upon hydrolysis.

An additional band around 60 kDa was seen in the Caiapo potato protein sample that was also resistant to pepsin, trypsin, and chymotrypsin digestion. This may be the same protein that Chen et al. (2009) found exhibited glutathione reductase activity and was resistant to trypsin and chymotrypsin digestion. A 60 kDa protein was not seen in the peel and blanched peel samples, but this was likely due to the concentration being below the staining threshold, not the absence of the protein. Caiapo potato protein exhibited higher solubility than the peel and blanched peel samples at pH = 2, and thus more proteins were able to be visualized.

### Trypsin and chymotrypsin inhibitory activity

Trypsin and chymotrypsin inhibitory activity was determined by monitoring the degradation of the pepsin band during incubation with trypsin and chymotrypsin. When trypsin and chymotrypsin were added to a pepsin solution, pepsin was hydrolyzed after incubation for 1 min under the conditions of the assay (data not shown). Trypsin inhibitors were present in the Caiapo, peel extract, and blanched peel extract after digestion with pepsin. Pepsin band density remained unchanged in the Caiapo potato protein and peel samples during incubation with trypsin (Figs. 2). Trypsin inhibitory activity was lower in the blanched peel sample than the Caiapo and peel samples. After 1 min of incubation with trypsin, pepsin band density was reduced to 90% of the original band density, and after 60 min, pepsin band density was reduced to 18% of the original density (Fig. 3). These results are consistent with previous studies that found heat treatment reduced the activity of sweet potato trypsin inhibitors (Obidairo and Akpochafo 1984; Zhang and Corke 2001; Kiran and Padmaja 2003). Trypsin inhibitors isolated from other sources have also been shown to exhibit resistance to pepsin digestion that is reduced by heat treatment. Liao et al. (2007) found that a trypsin inhibitor isolated from *Cassia obtusifolia* seeds, completely resistant to pepsin digestion before heating, became susceptible to pepsin digestion upon heating. Band density did not change in the nonheated sample after 60 min of incubation with pepsin; however, band density was reduced by 40% after 20 min of incubation with pepsin in the heated sample.

Chymotrypsin inhibitory activity did not appear to be present in any of the samples. The pepsin band was completely eliminated after 1 min of incubation with chymotrypsin in all samples and degradation products were visible (Figs. 3). While dual trypsin–chymotrypsin inhibitors have been identified in some plants (Gennis and Cantor 1976; Tasneem et al. 1996; Sasikiran et al. 2002, 2004; Tian et al. 2007), the sweet potato trypsin inhibitors do not appear to fall into this category, as trypsin inhibitory activity was present and chymotrypsin inhibitory activity was not present in the same samples. These results are consistent with Sugiura et al. (1973) who found that sweet potato trypsin inhibitors did not affect chymotrypsin activity.

### Amylase activity and amylase inhibitory activity

**Figure 5:**
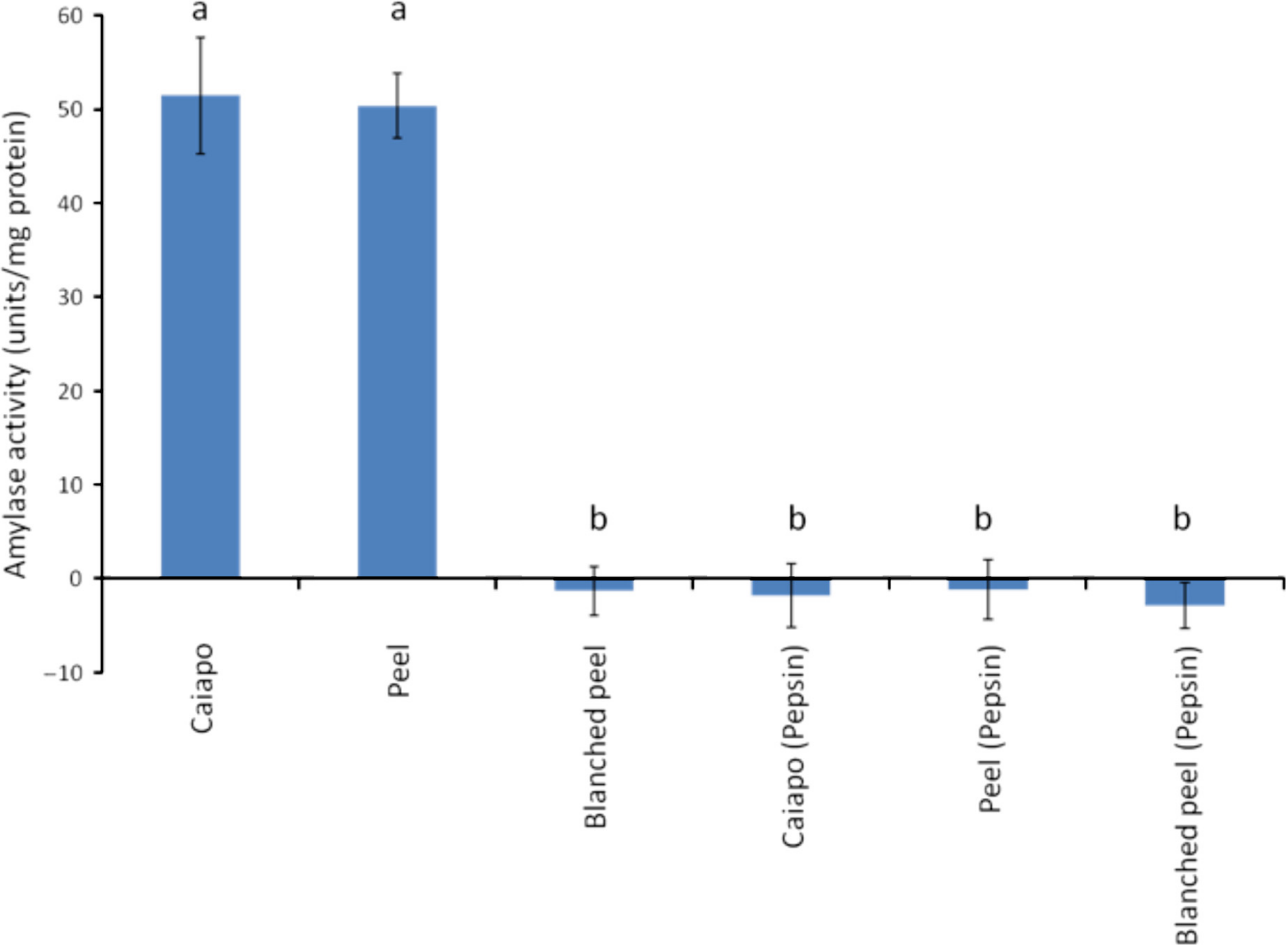
Amylase activity of Caiapo, peel, and blanched peel before and after incubation with pepsin for 1 h at pH = 2 and 37°C. Different letters represent statistically significant differences (*P* < 0.05).

**Figure 6:**
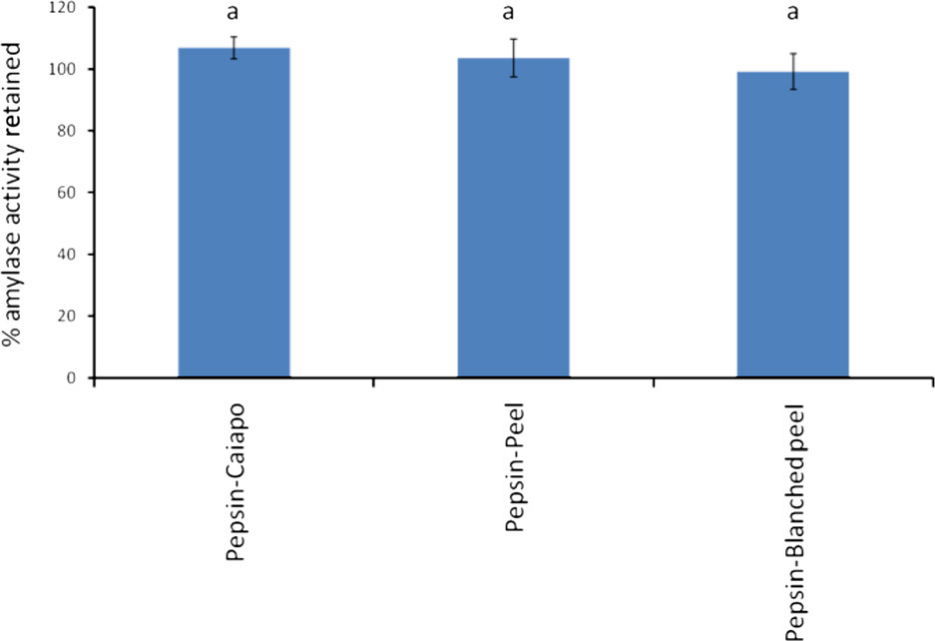
Activity of porcine pancreatic amylase (% retained) in the presence of Caiapo, peel, and blanched peel previously incubated with pepsin for 1 h at pH = 2 and 37°C. No statistically significant differences were observed (*P* < 0.05).

Caiapo and peel extract exhibited amylase activity; however, blanched peel extract did not exhibit amylase activity. These findings are in agreement with Hagenimana et al. (1994) who showed that sweet potato *α*-amylase and *β*-amylase were rapidly inactivated at high temperatures, such as those that would be used for blanching. The amylase activity of Caiapo and peel extract was eliminated by pepsin digestion (Fig. 5). The activity of porcine pancreatic amylase was not significantly changed by the presence of pepsin-digested Caiapo potato protein, peel extract, or blanched peel extract, indicating that amylase inhibitors were not present after pepsin digestion (Fig. 6).

Amylase inhibitors have been isolated from numerous plants, including legumes (Gibbs and Alli 1998), grains (Burgos-Hernández et al. 1999; Islamov and Fursov 2007), and tubers (Rekha et al. 1997, 1999; Sasikiran et al. 2002), as well as several types of seeds (Kotaru et al. 1992; Giri and Kachole 1998; Guzman-Partida et al. 2007), but their effects in vivo have been inconsistent. Carlson et al. (1983) found that despite high in vitro activity of a bean amylase inhibitor, in humans, glycemic response was not reduced by addition of the amylase inhibitor supplement to a high starch meal. Similarly, Bo-Linn et al. (1982) found that calories absorbed were not reduced by amylase inhibitor supplements given in conjunction with a high starch meal. Lack of stability to digestion was cited as a possible reason for the ineffectiveness of these supplements in vivo. Gibbs and Alli (1998) later found that an amylase inhibitor isolated from white kidney bean was resistant to pepsin digestion, but was readily hydrolyzed by trypsin and chymotrypsin. If amylase inhibitors were present in the sweet potato samples, they are likely susceptible to pepsin digestion, and thus would result in the same type of situation described above when consumed as a dietary supplement, that is, would not exhibit amylase inhibitory activity in vivo.

It is possible, however, that amylase inhibitors were not present in the sweet potato samples prior to digestion. Shivaraj et al. (1979) did not find amylase inhibitors in the sweet potatoes they tested and Rekha et al. (1999) only found amylase inhibitors in 79 of the 100 accessions they tested. The presence of native amylase activity in sweet potato protein complicates assays for amylase inhibitors. Shivaraj et al. (1979) overcame this issue by heating the extract for 10 min at 80°C to eliminate native amylase activity while Rekha et al. (1999) selectively precipitated the amylases with trichloroacetic acid prior to assaying for amylase inhibitory activity. As both of these methods could lead to inactivation or removal of amylase inhibitors as well as amylases, we attempted to take into account the native amylase activity in our calculations of amylase inhibitory activity by assaying for native amylase activity and porcine pancreatic amylase activity separately and then together, instead of inactivating or removing the native amylases. Expected activity was calculated by adding the native amylase activity and the porcine pancreatic amylase activity assayed separately, and then amylase inhibitory activity was determined by subtracting experimental amylase activity of the sweet potato protein and the porcine pancreatic amylase assayed together from the expected amylase activity. No amylase inhibitory activity was found in any of the extracts (data not shown) using this method. We decided to proceed with the digestion and then assay for amylase inhibition again after amylase activity was removed by digestion, as it has been suggested that the activities of *α*-amylase (like the porcine pancreatic amylase we used in the assay) and *β*-amylase (found in sweet potatoes) may not be additive (Delcour and Verschaeve 1987), which could have masked the presence of amylase inhibitors. This digestion method for eliminating native amylase activity was well-suited for our study because only the presence of digestion-resistant amylase inhibitors was important, as amylase inhibitors susceptible to digestion would be unlikely to exhibit nutraceutical effects in the body.

In conclusion, sweet potato peel contains a major storage protein, previously identified as sporamin that is resistant to simulated gastric digestion. Sweet potato peel protein functions as a trypsin inhibitor even after incubation with pepsin or trypsin. Amylase activity in peel was eliminated by heat and gastric digestion. Amylase inhibitor and chymotrypsin inhibitor activity was not present in peel, blanched peel or Caiapo potato protein after simulated gastric digestion.
